# Spectrum of underlying diseases in syncope and treatment of neurally-mediated syncope in children and adolescents over the past 30 years: A single center study

**DOI:** 10.3389/fcvm.2022.1017505

**Published:** 2022-11-28

**Authors:** Yaxi Cui, Ying Liao, Qingyou Zhang, Hui Yan, Ping Liu, Yuli Wang, Yan Sun, Wenrui Xu, Xueqin Liu, Junbao Du, Hongfang Jin

**Affiliations:** ^1^Department of Pediatrics, Peking University First Hospital, Beijing, China; ^2^Key Laboratory of Molecular Cardiovascular Sciences, Ministry of Education, Beijing, China

**Keywords:** syncope, neurally-mediated syncope, children, diagnosis, treatment, disease spectrum

## Abstract

**Background:**

Syncope is the primary cause of transient loss of consciousness, which causes severe physical and mental burdens to children and adolescents.

**Objective:**

The study was designed to analyze the spectrum of underlying diseases of syncope and treatment options for neurally-mediated syncope (NMS) in Chinese children and adolescents.

**Methods:**

Medical records including history, physical examination, blood biochemistry, standing test, head-up tilt (HUTT), sitting-up test, electroencephalogram (EEG), electrocardiogram (ECG), and echocardiography were retrospectively studied in children and adolescents admitted to the National Pediatric Syncope Center, Department of Pediatrics, Peking University First Hospital between 1992 and 2021. All the data were collected from the Beijing Kaihua Medical Management System (Kaihua, Beijing, China). Children who met the syncope diagnostic criteria were enrolled in the study. The spectrum of the underlying diseases of syncope in children and adolescents and the treatment options of NMS were analyzed.

**Results:**

A total of 1,947 children and adolescents with syncope were admitted, including 869 males (44.63%) and 1,078 females (55.37%) aged 1–18 years, with an average age of 11.1 ± 3.1 years. The number of children and adolescents with syncope displayed a gradually increasing trend between 1992 and 2021 except after 2020. NMS proportion increased, and the proportion of unexplained syncope decreased (χ^2^ = 128.839, *P* < 0.01). The treatment options of NMS mainly included autonomic nervous function exercise (549, 34.46%), oral rehydration salt (ORS; 445, 27.94%), metoprolol (219, 13.75%), midodrine (120, 7.53%), ORS plus metoprolol (139, 8.73%), ORS plus midodrine (120, 7.53%), and pacemakers (1, 0.06%). Patients with vasovagal syncope (VVS) coexisting with postural orthostatic tachycardia syndrome (POTS) were more likely to take pharmacological treatments than those with VVS or POTS only (χ^2^ = 41.696, *P* < 0.01).

**Conclusion:**

The number of children with syncope displayed an increasing trend before 2020, and the proportion of unexplained syncope decreased. Autonomic nervous function exercise was the most common treatment for children and adolescents with NMS. Children with VVS coexisting with POTS were more likely to receive pharmacological treatments than those with either.

## Introduction

Syncope is the main cause of transient loss of consciousness. Syncope often occurs rapidly, with temporary and spontaneously recoverable loss of consciousness and muscle tone due to brain hypoperfusion (1). A previous study reported that 1% of patients with syncope in hospital visits (2) and 15% of children and adolescents before the age of 18 have experienced at least one syncopal episode (3). Syncope can cause accidental injuries, mental illnesses such as anxiety, or death, imposing a serious burden on families and children and reducing the quality of life (4–6). There are various causes of syncope, including neurally-mediated syncope (NMS), cardiac syncope (CS), and unexplained syncope. NMS includes vasovagal syncope (VVS) and postural orthostatic tachycardia syndrome (POTS) as its main underlying diseases (7). In 2020, our team reported sitting intolerance (SI) in children for the first time, including sitting tachycardia syndrome (STS) and sitting hypertension (SHT) (8), expanding the spectrum of syncope in children and adolescents. This study retrospectively analyzed the spectrum of underlying diseases in syncope in children and adolescents and described the treatment options for children and adolescents with NMS to increase the understanding and awareness of pediatric syncope and provide help for NMS treatment in children and adolescents.

## Materials and methods

### Study participants

Between January 1992 and December 2021, 1,947 children and adolescents with syncope were admitted to the National Pediatric Syncope Center, Department of Pediatrics, Peking University First Hospital, including 869 males (44.63%) and 1,078 females (55.37%), aged 1–18 years (average age, 11.1 ± 3.1 years). All data were collected from Beijing Kaihua Medical Management System (Kaihua, Beijing, China). The Ethics Committee of Peking University First Hospital approved the study. A waiver of informed consent was granted.

### Diagnostic procedures

Comprehensive clinical and family histories and physical examinations were required for all children and adolescents with syncope (9). The standing test and electrocardiography (ECG) were performed. Cardiomyopathy, congenital heart disease (CHD), pulmonary hypertension, arrhythmia, and other CS could be identified by further appropriate tests, such as echocardiography, Holter ECG, cardiac catheterization, angiocardiography, electrophysiological, and genetic test examinations. When the etiology of syncope remained unidentified, the head-up tilt test (HUTT) was used for further diagnosis.

#### Standing test

The patients were asked to lay in a supine position for 10 min and keep upright for another 10 min in a quiet environment. The baseline heart rate (HR), blood pressure (BP), and ECG of children were dynamically observed during this period. The standing test assisted the diagnosis of POTS, orthostatic hypotension (OH), and orthostatic hypertension (OHT) (7).

#### Head-up tilt test

All drugs that affected autonomic nerve function were discontinued at least five half-lives before the test. The children fasted 4 h before HUTT. The test environment was quiet and dimly lit and at an appropriate temperature. The children were required to lie on a tilt bed in a supine position for 10–30 min and then in a tilted position at 60° for 45 min. BP, HR, and ECG were recorded continuously. The tilt position was maintained for 45 min until the children responded positively. VVS diagnosis and its hemodynamic subtypes, POTS, OH, and OHT, were identified using HUTT (7). If necessary, sublingual nitroglycerin-provoked HUTT assisted in diagnosis. After sublingual administration of nitroglycerin 4–6 μg/kg (maximum ≤ 300 μg), children were asked to maintain the same position for another 20 min. BP, HR, and ECG of children were continuously monitored for 20 min until a positive reaction occurred (10).

#### Sitting-up test

The test was conducted in a quiet room at a comfortable temperature. A supine rest for 10 min was needed to maintain a stable HR and BP was measured using the Finapres Medical System-FMS (FinometerPRO, FMS, Netherlands). The children were asked to sit upright in a chair with knees bent 90°, feet touching the floor, hands hanging down naturally, and back leaning against nothing for 10 min. During this period, HR, BP, and intolerance symptoms of children and adolescents were noted. The sitting-up test assisted in diagnosing STS and SHT (8).

### Diagnostic criteria

#### Diagnostic criteria of VVS

The diagnostic criteria of VVS used in this study were: (1) a history of syncope; (2) predisposing syncope factors; (3) a positive hemodynamic response during HUTT; and (4) absence of metabolic, neurological, or structural cardiovascular diseases. A positive response to HUTT was determined by children and adolescents with syncope or presyncope responding with one of the following conditions: (1) decreased BP; (2) decreased HR; (3) sinus arrest and premature junctional contractions; or (4) atrioventricular block or cardiac arrest ≥3 s. The standard BP was considered decrease when systolic BP was ≤80 mmHg, diastolic BP ≤50 mmHg, or a 25% of BP reduction. The HR drop was considered when HR <75 beats/min (4–6 years old), <65 beats/min (6–8 years old), and <60 beats/min (over 8 years old) (7, 11, 12).

#### Diagnostic criteria of POTS

The POTS diagnostic criteria used in this study were: (1) predisposing factors; (2) orthostatic intolerance (OI) symptoms such as dizziness, headache, blurred vision, chest tightness, palpitations, fatigue, or syncope; (3) a positive response during a standing test or HUTT; and (4) absence of metabolic, neurological, or structural cardiovascular diseases. The positive criteria for the standing test or HUTT were identified when an increase in HR by ≥ 40 beats/min after 10 min of standing upright or maximum HR ≥130 beats/min (6–12 years old) or ≥125 beats/min (13–18 years old) appeared (7, 11, 12).

#### Diagnostic criteria of OH and OHT

The diagnostic criteria of OH and OHT used in this study were: (1) predisposing factors; (2) syncope, dizziness, vertigo, pale complexion, fatigue, blurred vision, chest tightness, palpitations, abdominal pain, nausea, or vomiting as OI symptoms after prolonged upright standing; (3) positive response during a standing test or HUTT; and (4) absence of metabolic, neurological, or structural cardiovascular diseases. The positive criteria of OH for the standing test or HUTT were as follows: systolic BP decreased by ≥20 mmHg or diastolic BP decreased by ≥10 mmHg during the initial 3 min of standing test or HUTT, with no significant HR change. The positive criteria of OHT for the standing test or HUTT were as follows: systolic BP increased by ≥20 mmHg or diastolic BP increased by ≥10 mmHg during the initial 3 min of the standing test or HUTT (7, 11, 12).

#### Diagnostic criteria of STS and SHT

The diagnostic criteria of STS used in this study were: (1) predisposing factors associated with prolonged sitting or position changes; (2) syncope, dizziness, pale complexion, fatigue, chest tightness, nausea, palpitations, abdominal pain, or vomiting as SI symptoms after prolonged upright sitting or the sudden position change from supine to sitting; and (3) positive response during a sitting-up test. The positive criteria of STS were as follows: HR increased by ≥25 beats/min within the initial 3 min of the sitting position. The positive criteria of STS were as follows: BP increased by ≥20/20 mmHg within the initial 3 min of sitting (8).

### Treatment options for NMS in children and adolescents

The treatment options for children and adolescents with NMS included non-pharmacological and pharmacological treatments (7, 11, 12).

#### Non-pharmacological treatments

Non-pharmacological treatment included the exercise of autonomic function and increased intake of water and salt. The exercise of autonomic nervous function mainly included upright training and wiping of limbs with a dry towel. In upright training, children were asked to stand against a wall at home with feet 15 cm away. The standing time was based on the children's tolerance and gradually extended (the longest standing time for each upright training was 30 min) 1–2 times a day. Children were accompanied by family members when doing upright training to prevent injuries caused by syncope or presyncope. Oral rehydration salt (ORS; 5.125 g/bag) of one bag per day was recommended, with the contents of each bag being dissolved in 250–500 ml of water, which could supplement the water and salt intake.

#### Pharmacological treatments

Pharmacological treatments included beta-blockers, alpha-receptor agonists, and pacemakers, specifically (1) metoprolol (AstraZeneca, London, UK): 0.5 mg/kg/day, orally with HR being self-monitored during administration and (2) midodrine (Sinopharm Chuankang, Chengdu, China): 2.5 mg/d orally.

#### Pacemaker therapy

Children with VVS had recurrent syncope accompanied by a prolonged cardiac arrest >4 s, and chamber pacing was recommended to reduce syncope recurrence (7).

### Statistical analyses

The data were analyzed using SPSS 25.0 software (IBM Corporation, New York, USA). The normality of data was assessed using the Shapiro–Wilk test. Continuous data were expressed as means ± standard deviations, and categorical data were expressed as frequencies (percentages). The intergroup differences were compared using the Chi-squared test. Bonferroni adjustment was used for the *post hoc* test following the Chi-squared test. *P* < 0.05 indicated that the difference was significant.

## Results

### Demographic data for children and adolescents with syncope

Among 1,947 children and adolescents, 101 had syncope between 1992 and 2001, including 44 males (43.56%) and 57 females (56.44%), aged 3–17 (10.7 ± 3.1) years. A total of 374 children and adolescents with syncope were admitted between 2002 and 2011, including 169 males (45.19%) and 205 females (54.81%) aged 1–18 (11.1 ± 3.3) years. A total of 1,472 children and adolescents with syncope were admitted between 2012 and 2021, including 656 males (44.57%) and 816 females (55.43%) aged 1–18 (11.2 ± 3.1) years (Table 1).

**Table 1:** Demographic data of children with syncope over three decades.

**Period**	**Sex (M/F, *n*)**	**Age (year)**	**Total (*n*)**
1992–2001	44/57	10.7 ± 3.1	101
2002–2011	169/205	11.1 ± 3.3	374
2012–2021	656/816	11.2 ± 3.1	1,472

### Number of children and adolescents with syncope

**Figure 1:**
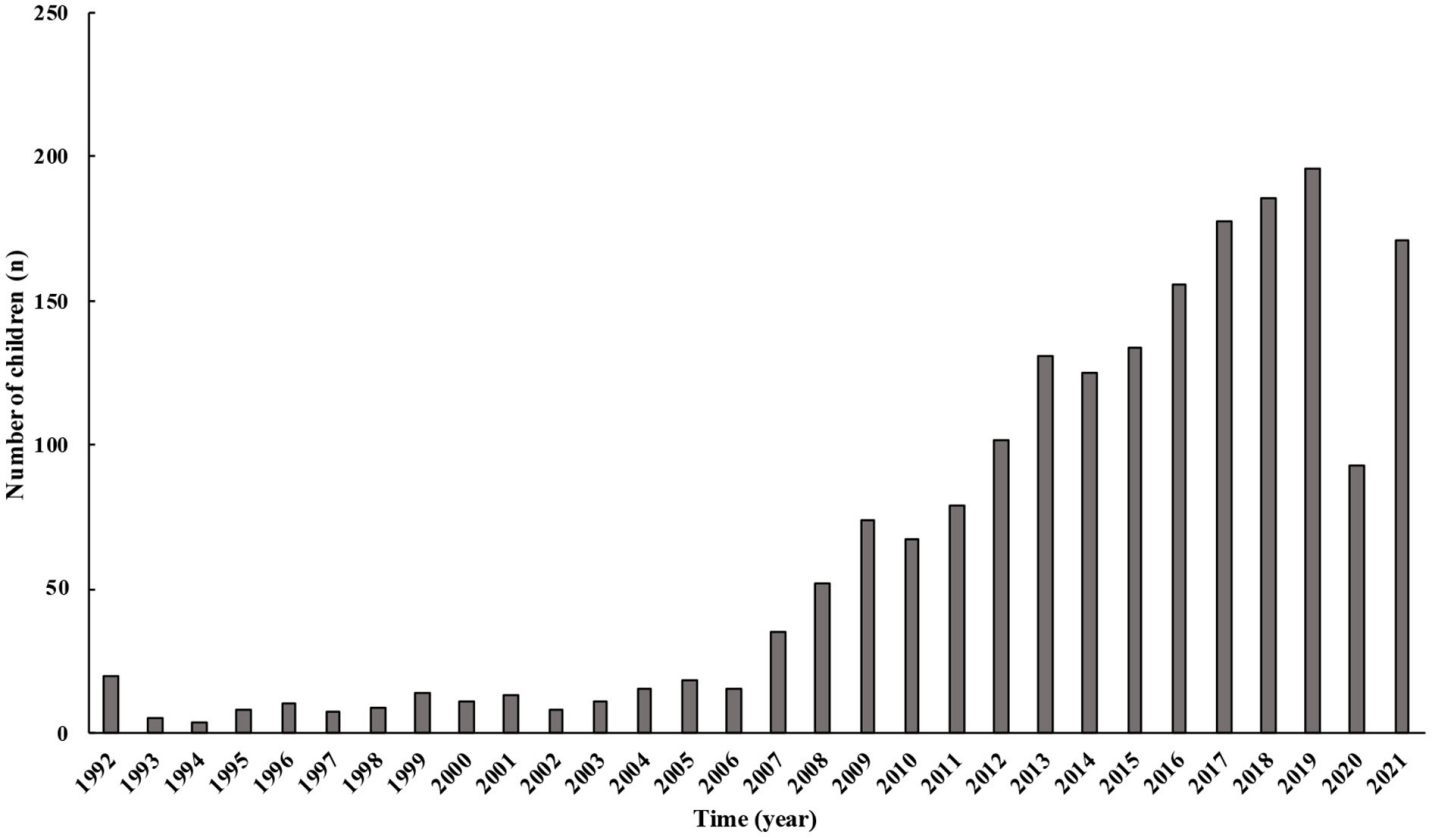
Changes in the number of children with syncope over years. From 1992 to 2021, the number of patients admitted to our center in China gradually increased. The number of patients after 2020 was progressively lower than before.

The number of children and adolescents with syncope gradually increased except after 2020. Between 1992 and 2005, the growth in the number of children was more moderate during the first 15 years. Since 2006, an increasing prevalence of syncope in children has been observed, which peaked in 2019, followed by a significant decrease in 2020 (Figure 1).

### Disease spectrum of children and adolescents with syncope

The proportion of syncopal underlying diseases changed significantly between 1992 and 2021. Between 1992 and 2001, NMS accounted for 46.54% (47/101), CS for 8.91% (9/101), and unexplained syncope for 44.55% (45/101) of the cases. Between 2002 and 2011, NMS accounted for 76.20% (285/374), CS for 6.95% (26/374), and unexplained syncope for 16.85% (63/374) of the cases. Furthermore, between 2012 and 2021, NMS accounted for 85.67% (1,261/1,472), CS for 5.50% (81/1,472), and unexplained syncope for 8.83% (130/1,472) of the cases. In the past 30 years, the NMS proportion gradually increased, while the proportion of unexplained syncope gradually decreased. This change in disease spectrum proportions was significant (χ^2^ = 128.839, *P* < 0.01).

### NMS spectrum

Between 1992 and 2001, the NMS spectrum was mainly composed of VVS, OH, and micturition syncope; the highest proportion of NMS was VVS (93.62%). Between 2002 and 2011, the NMS spectrum comprised VVS, POTS, OH, VVS coexisting with POTS, and micturition syncope. The highest proportion of NMS was POTS (69.12%), followed by VVS (24.21%). Between 2012 and 2021, the NMS spectrum included VVS, POTS, OH, OHT, VVS coexisting with POTS, POTS coexisting with OHT, POTS coexisting with STS, VVS coexisting with OHT, VVS coexisting with STS, VVS coexisting with POTS and STS, and POTS coexisting with STS and SHT. The highest proportion of NMS was VVS (40.60%), followed by POTS (30.85%; Table 2).

**Table 2:** Changes in neurally-mediated syncope spectrum over three decades.

**Disease category**	1992–2001*	2002–2011*	2012–2021*
VVS, *n* (%)	44 (93.62%)	69 (24.21%)	512 (40.60%)
OH, *n* (%)	1 (2.13%)	5 (1.76%)	13 (1.03%)
POTS, *n* (%)	/	197 (69.12%)	389 (30.85%)
OHT, *n* (%)	/	/	55 (4.36%)
VVS + POTS, *n* (%)	/	10 (3.51%)	191 (15.15%)
POTS + OHT, *n* (%)	/	/	39 (3.09%)
POTS + STS, *n* (%)	/	/	16 (1.27%)
VVS + OHT, *n* (%)	/	/	12 (0.95%)
VVS + STS, *n* (%)	/	/	6 (0.48%)
VVS + POTS + STS, *n* (%)	/	/	14 (1.11%)
POTS + STS + SHT, *n* (%)	/	/	4 (0.32%)
Micturition syncope, *n* (%)	2 (4.25%)	4 (1.40%)	10 (0.79%)
Total	47	285	1,261

VVS, vasovagal syncope POTS; postural orthostatic tachycardia syndrome; OHT, orthostatic hypertension; OH, orthostatic hypotension; STS, sitting tachycardia syndrome; SHT, sitting hypertension; +, coexisting diseases; ^*^, year.

### CS spectrum

Between 1992 and 2021, arrhythmias accounted for most children with CS being admitted to our center (101/116), followed by CHD (3/116), including congenital mitral valve insufficiency, congenital aortic stenosis, and tetralogy of Fallot. Other CS diseases consisted of anomalous origin of the coronary artery (2/116), cardiomyopathy (6/116), and pulmonary arterial hypertension (4/116).

### Treatment of NMS

The treatment options for NMS mainly included autonomic nervous function exercise (549, 34.46%), ORS (445, 27.94%), metoprolol (219, 13.75%), midodrine (120, 7.53%), ORS plus metoprolol (139, 8.73%), ORS plus midodrine (120, 7.53%), and pacemakers (1, 0.06%).

In addition, for children and adolescents with VVS, the treatment option with the highest proportion was autonomic nervous function exercise (38.97%), followed by ORS (29.04%) and metoprolol (12.73%). For children and adolescents with POTS, the largest proportion of the treatment options was autonomic nervous function exercise (31.75%), followed by ORS (29.81%) and metoprolol (14.11%). For children and adolescents with POTS coexisting with VVS, the treatment option with the largest proportion was ORS (23.38%), followed by autonomic nervous function exercise (19.40%) and ORS plus metoprolol (19.41%; Table 3). There were significant differences in the proportions of treatment options among the three groups (χ^2^ = 41.696, *P* < 0.01). *Post hoc* analysis revealed that children with VVS coexisting with POTS were more likely to take pharmacological medication than did those with either (*P* < 0.05). However, no significant difference was observed in the proportion of treatment options between patients with POTS and those with VVS.

**Table 3:** The proportions of treatment options in children with VVS, POTS, and VVS coexisting with POTS.

**Treatment option**	**VVS, *n* (%)**	**POTS, *n* (%)**	**VVS + POTS, *n* (%)**
**Non-pharmacological treatment**	438 (68.01%)	349 (61.56%)	86 (42.78%)
Autonomic nervous function exercise	251 (38.97%)	180 (31.75%)	39 (19.40%)
ORS	187 (29.04%)	169 (29.81%)	47 (23.38%)
**Pharmacological treatments**	205 (31.83%)	218 (38.44%)	115 (57.22%)
Metoprolol	82 (12.73%)	80 (14.11%)	25 (12.44%)
Midodrine	41 (6.37%)	48 (8.46%)	23 (11.44%)
ORS plus metoprolol	31 (4.81%)	58 (10.23%)	39 (19.41%)
ORS plus midodrine	51 (7.92%)	32 (5.64%)	28 (13.93%)
**Pacemaker therapy**	1 (0.16%)	/	/
**Total**	644	567	201

VVS, vasovagal syncope; POTS, postural orthostatic tachycardia syndrome; ORS, oral rehydration salt; +, coexisting diseases.

## Discussion

In this study, we analyzed the status and changes of the underlying disease spectrum and treatment options of pediatric syncope in our center in the past 30 years between 1992 and 2021. We observed that the number of patients with syncope gradually increased except after 2020. The proportion of unexplained syncope significantly decreased [44.55% (between 1992 and 2001) vs. 16.84% (between 2002 and 2011) vs. 8.83% (between 2012 and 2021), *P* < 0.01]. In the past 10 years (2012–2021), the spectrum of children and adolescents with syncope also expanded. NMS is the major underlying disease of patients with syncope and accounts for 85.67% of syncope cases.

William R. Gowers first described “vagal” symptoms in 1907, and Thomas Lewis later redefined “vasovagal syncope” in 1932, which stimulated a new age of syncope understanding (13). However, before the 20^th^ century, pediatricians' awareness and understanding of syncope in children and adolescents was insufficient. With the VVS diagnosis in Chinese children in the 1990s by our team, this disease has been increasingly understood by Chinese children and their families (14). VVS affects children's learning and quality of life and burdens children and their parents. An increasing number of children and adolescents with syncope were accurately diagnosed and treated in the Pediatric Syncope Clinic of Peking University First Hospital. This survey revealed that the number of children and adolescents with NMS increased gradually with slight fluctuations. This may be related to the standardization of diagnostic procedures (9), the heavy learning burden for students, and health awareness. The proportion of CS was 5.50%−8.91% in different decades, which might be underestimated in this study. The syncope guidelines of ACC indicated that psychogenic accounted for 8%−15% of cases (12, 15). However, Courtheix et al. (16) described 5.1% of CS in children with syncope in a single center. This may be because the single-center study could not represent the incidence and spectrum of the underlying diseases of syncope. Our study revealed that the number of children and adolescents with syncope in hospitals decreased significantly in 2020. The COVID-19 pandemic was the main reason for a decrease in the number of patients with syncope who visited the hospitals. Additionally, given that the main group of syncope in children comprised students, implementing online classes for students reduced syncopal risks, such as a stressful environment and prolonged standing (17).

In 1997, our team reported VVS in children for the first time in China (14) and suggested that HUTT could assist in identifying the underlying syncope diseases in children. Therefore, with HUTT awareness (18), the number of children and adolescents with unexplained syncope has gradually decreased. In 2012, our team proposed the diagnostic criteria for pediatric OHT through epidemiological studies. We also established the diagnostic criteria for POTS according to the epidemiological investigation in China in 2015 (19), contributing to POTS diagnosis and management in children worldwide. Recently, we proposed SI in children for the first time in 2020, including STS and SHT (8). Therefore, the syncope spectrum was gradually enlarged, and the proportion of unexplained syncope in children decreased over this period (χ^2^ = 128.839, *P* < 0.01).

Our study revealed that different NMS subtypes might coexist. Studies have indicated that VVS and POTS are different diseases that need to be distinguished by clinical experience (20). However, Cai et al. proved that tachycardia existed in POTS children when they changed from a supine to a sitting position (21). Furthermore, an increase of HR ≥ -22 beats/min within 10 min could predict this POTS diagnosis, which proved that POTS and SI subtypes could coexist. Skerk et al. also reported a case in a female with a positive POTS response followed by VVS in HUTT (22). However, NMS pathogenesis is not well understood. It may be associated with abnormal baroreflex sensitivity (BRS), hypovolemia, vasomotor dysfunction, and autonomic dysfunction (23). Baroreceptors in the carotid sinus and aortic arch are essential in regulating the cardiovascular system with position changes. Studies have revealed that patients with VVS, POTS, or OH have an abnormal BRS (24). The change from a supine to an upright position can activate the renin-angiotensin-aldosterone system, leading to vasoconstriction and increased blood volume (25). Patients with VVS, OH, or OHT have experienced increased renin, angiotensin, and aldosterone (26). Dysfunction of the autonomic nerve is the primary pathogenesis of patients with NMS. In addition, heart rate variability, reflecting autonomic nervous function, is also abnormal to varying degrees in patients with VVS and POTS (27). As previously mentioned, complex pathophysiological mechanisms may be responsible for the possible coexistence of different NMS subtypes. Additionally, studies have suggested that the coexistence of different diseases in patients is vital to treatment selection. For instance, in the early stage of positive VVS, the appearance of POTS hemodynamic characteristics suggested that sympathetic hyperactivation was involved in the VVS mechanism, which advised the use of β-receptor blockers (28).

In addition to investigating the syncope spectrum changes, this study also focused on the treatment options for children with NMS. Among them, the largest proportion was autonomic nervous function exercise, per the guidelines' principle of preferred non-pharmacological treatment recommendations (7). According to our study, children with VVS coexisting with POTS were likely to be prescribed pharmacological treatment compared with other types of NMS. There may have been interactions in NMS pathogenesis; therefore, pediatricians were inclined to treat using medications. However, insufficient blood volume, increased sympathetic activity, high catecholamines, excessive vasodilation, and other factors are involved in the complex pathogenesis of NMS (23). Therefore, treatment options should target these mechanisms. For instance, ORS might be considered for those with insufficient blood volume (29, 30), metoprolol for those with increased sympathetic activity and high catecholamines (31), and midodrine for those with excessive vasodilation, in their main mechanisms, respectively (32). The pathogenesis of patients with NMS is diverse (33); therefore, NMS treatments should require an individualized evaluation by pediatricians (34).

There were, however, some limitations to the study. First, it was a single-center study in China. The patients came from different parts of China; nonetheless, multicenter studies are still needed to clarify the spectrum of the underlying diseases in syncope. In addition, the number of cases analyzed by this study were limited. However, the study results may significantly improve the understanding of the spectrum of underlying diseases in syncope and its changing trend in China, increase pediatricians' awareness of syncope, and provide a general profile of the treatment options for syncope in children and adolescents.

## Data availability statement

The raw data supporting the conclusions of this article will be made available by the authors, without undue reservation.

## Ethics statement

The studies involving human participants were reviewed and approved by the Ethics Committee Peking University First Hospital. Written informed consent for participation was not provided by the participants' legal guardians/next of kin because the required informed consent was permitted to be waived by the Ethics Committee Peking University First Hospital.

## Author contributions

YC collected and analyzed data, drafted initial manuscript, interpreted the result, and critically revised the manuscript. YL, QZ, HY, YS, WX, and XL collected data and revised the manuscript. PL and YW performed the head-up tilt test and reviewed the manuscript. JD and HJ conceptualized and designed the study, and critically reviewed and revised the manuscript. All authors agreed to accept responsibility for this work and agreed the final manuscript as submitted.

## Funding

This study was supported by the National High Level Hospital Clinical Research Funding (Multi-center Clinical Research Project of Peking University First Hospital) (2022CR59), the Clinical Medicine Plus X-Young Scholars Project of Peking University, and the Fundamental Research Funds for the Central Universities (PKU2022LCXQ028).

## Conflict of interest

The authors declare that the research was conducted in the absence of any commercial or financial relationships that could be construed as a potential conflict of interest.

## Publisher's note

All claims expressed in this article are solely those of the authors and do not necessarily represent those of their affiliated organizations, or those of the publisher, the editors and the reviewers. Any product that may be evaluated in this article, or claim that may be made by its manufacturer, is not guaranteed or endorsed by the publisher.
